# Phytochemical and Pharmacological Profiling of *Heritiera fomes* Buch. Ham. Deciphered Thrombolytic, Antiarthritic, Anthelmintic, and Insecticidal Potentialities via In Vitro Approach

**DOI:** 10.1155/2022/2594127

**Published:** 2022-07-18

**Authors:** Farhana Alam Ripa, Md. Jamal Hossain, Mst. Luthfun Nesa, Miss Sharmin Zahan, Saikat Mitra, Mohammad A. Rashid, Arpita Roy, Saad Alghamdi, Mazen Almehmadi, Osama Abdulaziz

**Affiliations:** ^1^Department of Pharmacy, Brac University, 41-Pacific Tower, Mohakhali, Dhaka 1212, Bangladesh; ^2^Department of Pharmacy, State University of Bangladesh, 77 Satmasjid Road, Dhanmondi, Dhaka 1205, Bangladesh; ^3^Department of Pharmacy, Faculty of Pharmacy, University of Dhaka, Dhaka 1000, Bangladesh; ^4^Department of Pharmaceutical Chemistry, Faculty of Pharmacy, University of Dhaka, Dhaka 1000, Bangladesh; ^5^Department of Biotechnology, School of Engineering & Technology, Sharda University, Greater Noida, India; ^6^Laboratory Medicine Department, Faculty of Applied Medical Sciences, Umm Al-Qura University, Makkah, Saudi Arabia; ^7^Clinical Laboratory Sciences Department, College of Applied Medical Sciences, Taif University, Taif, Saudi Arabia

## Abstract

Medicinal plants have been crucial in treating various chronic ailments since ancient times. The objective of this study was to evaluate in vitro pharmacological properties of petroleum ether, chloroform, and ethyl acetate soluble fractions of ethanolic extract (leaf, bark, and root) of *Heritiera fomes* Buch. Ham., including the phytochemical screening of the plant. Thrombolytic and antiarthritic properties were assessed through the clot lysis and protein denaturation experimental method, correspondingly. Anthelmintic and insecticidal activities were studied against *Pheretima posthuma* and *Tribolium castaneum*, respectively. The phytochemical analysis exhibited numerous active phytochemicals in different solvent fractions. In thrombolytic investigation, among all crude extracts, ethanolic leaf extract showed the highest 33.12 ± 7.52% clot lysis as compared to standard streptokinase (67.77 ± 9.78%). In antiarthritic assay, all the tested samples exhibited noteworthy protein denaturation in dose-dependent manner (100–500 *μ*g/mL), whereas the utmost percentage inhibition was noticed for chloroform extract of roots (63.28 ± 5.96% at 500 *μ*g/mL). All crude extracts exhibited a significant anthelmintic activity in different concentrations (25–75 mg/mL) and revealed paralysis and death of earthworms in comparison with albendazole; ethanolic extract of the bark was found to be more potent at the highest dose. For the insecticidal test, ethanolic extract of the leaf showed the utmost mortality rate (73%). The outcomes of the investigation confirmed the potential thrombolytic, antiarthritic, anthelmintic, and insecticidal activities of the different extracts of *H. fomes*, and hence, advanced studies on the isolation and identification of active phytocompounds are highly needed for new drug development.

## 1. Introduction

Nature is often a brilliant sign to point out the distinguished marvels of existence. Natural merchandise from plants, animals, and minerals area unit the premise for treating numerous human diseases [1, 2]. Herbs, particularly medicative herbs, have perpetually acted as an associate overall indicator of scheme health [3]. According to the World Health Organization (WHO), 65–85% of the world's population still depends on plants as a resource for primary healthcare [4–6]. Plant-derived remedies function as a model to develop more efficient and less noxious medicines. The healing properties of medicinal plants are possibly owing to the existence of miscellaneous secondary metabolites like alkaloids, glycosides, flavonoids, phenols, saponins, and sterols [7–9]. There is an escalating attention in correlating the phytochemicals of a medicinal plant with its medicinal property [10, 11].

Bangladesh possesses a rich flora and expanded genetic resources of medicinal plants [12–14]. Reports proved that in vitro screening approaches could offer the required preliminary remarks necessary to choose crude plant extracts with potentially valuable properties for advanced chemical and pharmacological investigations [15, 16]. This information exhilarated us to find out new hope for medical science from *Heritiera fomes* Buch. Ham. plant. It is an important moderate size mangrove tree growing copiously in the Sundarbans [17, 18]. The earlier research showed that *H. fomes* contains alkaloids, glycosides, flavonoids, saponins, carbohydrates, phenols, gums, and sterols, which were confirmed during their phytochemical investigations of different plant parts extracts [17, 19]. The plant is used in different types of gastrointestinal and skin disorders, diabetes, goiter, and cardiovascular diseases. It also possesses wound healing, antioxidant, antinociceptive, antimicrobial, anticancer, and insect-repelling properties [17, 19–21].

Although few studies were conducted earlier on the ethnomedicinal properties of *H. fomes,* no record has been found on its in vitro thrombolytic, antiarthritic, anthelmintic, and insecticidal activities. Therefore, the study was conducted to assess the in vitro thrombolytic, antiarthritic, anthelmintic, and insecticidal properties of ethanolic extract and its three different partitions (ethyl acetate, petroleum ether, and chloroform) of leaf, bark, and root of *H. fomes* along with the phytochemical screening with a view to exploring its possible applications in pharmaceutics.

## 2. Materials and Methods

### 2.1. Plant Materials

Fresh leaves, barks, and roots of *H. fomes* were collected from the Sundarbans, Bagerhat district, Bangladesh, in December 2020. The plant was identified and authenticated by a taxonomist of Bangladesh National Herbarium, Mirpur, Dhaka (DACB Accession No: 50664), and was preserved in our laboratory for future reference. The collected plant parts were washed cautiously with running tap water to remove dirt and then rinsed with distilled water. The leaves, barks, and roots were separated, cut into small pieces, shade dried for two weeks, and pulverized into a coarse powder with a laboratory electric blender. The powdered plant materials were stored separately in airtight containers in a cool, dark, and dry place for further use.

### 2.2. Chemicals and Reagents

All the reagents were analytical reagent grades procured from Sigma Chemical Co. (St. Louis, MO, USA) and Merck (Darmstadt, Germany). 5 ml phosphate buffered saline was added to the commercially available lyophilized streptokinase (15,00,000 IU) vial (S-kinase, Popular Pharmaceuticals Ltd., Bangladesh) and mixed accurately. The concentration of the streptokinase was accustomed to being 30,000 IU and used as the reference standard for thrombolytic activity. Diclofenac sodium and albendazole were collected from Square Pharmaceutical Ltd., Bangladesh.

### 2.3. Extraction Procedure

The powered plant materials of leaf, bark, and root (500 gm) were taken into three different clean glass containers and soaked in 2 L ethanol for a week with random shaking and stirring. The mixture was then individually filtered by Whatman filter paper (number 1) into three different clean beakers. The filtrate was concentrated using a rotary evaporator at 40°C under reduced pressure. Around 5 g of the concentrated extracts (leaf, bark, and root) of *H. fomes* was subjected to solvent-solvent partitioning following the modified Kupchan partitioning procedure [22] into petroleum ether, chloroform, and ethyl acetate soluble portions and were individually assessed for phytochemical and in vitro biological activities. The ethanol, petroleum ether, chloroform, and ethyl acetate extracts of the leaf were tagged as LE, LPE, LC, and LEA, respectively. Bark extracts were labeled as BE (ethanolic), BPE (petroleum ether), BC (chloroform), and BEA (ethyl acetate); whereas, root extracts were marked as RE, RPE, RC, and REA for ethanol, petroleum ether, chloroform, and ethyl acetate solvents, correspondingly.

### 2.4. Phytochemical Analysis

The freshly prepared crude extracts were qualitatively tested for the presence of active bioactive phytochemicals like carbohydrates, alkaloids, glycosides, flavonoids, saponins, sterols, tannins, fixed oil, resins, and phenols by using standard procedures [23].

### 2.5. Thrombolytic Activity

In vitro thrombolytic potentiality of the experimented extracts was evaluated by applying the method described by Tabassum et al. [24]. Venous blood samples (4 mL) were collected from healthy human volunteers (*n* = 10) with no hematological disorders or with any history of taking anticoagulant therapy. Then, the collected blood sample was transferred to several preweighed sterile microcentrifuged tubes (1 mL/tube). Informed written consent was taken from each volunteered blood donor. One mL of blood was transferred to each preweighted sterile Eppendorf tube and incubated at 37°C for 45 minutes. The serum was completely eradicated after clot formation. Each tube having a clot was further weighted to measure the clot weight (clot weight = weight of clot containing tube−weight of tube alone). Then, 100 *μ*L of different crude extracts was added to each of these tubes separately. Here, we used streptokinase and distilled water as positive control and negative control, respectively. Then, 100 *μ*L of streptokinase and 100 *μ*L of distilled water were individually added to the control marked Eppendorf tubes. All the tubes were then incubated at 37°C for 90 minutes and observed for clot lysis. After incubation, the released fluid was discarded, and tubes were again weighted to see the difference in weight after clot disruption. The difference obtained in weight taken before and after clot lysis was represented as a percentage of clot lysis. The experiment was done three times with different blood samples of volunteers. We have used the following formula to calculate the % of clot lysis [24]:

(1) $$\begin{array}{c} \%\text{ clot lysis}=\left(\frac{\text{Weight of the clot lysis}}{\text{Weight of clot before lysis}}\right)\times 100\text{.} \end{array}$$

### 2.6. Antiarthritic Activity

To assess antiarthritic activity, we followed the method described by Naz et al. [25]. At first, 0.45 ml bovine serum albumin (5% aqueous solution) and 0.05 ml of different experimented extracts at various concentrations (100–500 *μ*g/mL) were mixed together. Then, all the samples were incubated at 37 C for 30 minutes and further heated at 57°C for 3 minutes to induce denaturation of protein. Later, 2.5 mL phosphate buffer (pH 6.3) was added to each tube after cooling them to room temperature and absorbance of turbidity was measured at 660 nm. Instead of the extract, we used 0.05 mL distilled water solution as control. In this experiment, diclofenac sodium was used as standard at the same concentrations as the crude extracts and treated similarly. The result was calculated by using the following formula:

(2) $$\begin{array}{c} \%\text{inhibition}=\frac{\text{Vc}-\text{Vt}}{\text{Vt}}\times 100, \end{array}$$

where Vt is the absorbance of test samples; Vc is the absorbance of control.

### 2.7. Anthelmintic Activity

The anthelmintic activity of *H. fomes* extracts was assayed by the modified Gopalakrishnan et al.' [26] method. Here, we used Bangladeshi earthworms (*Pheretima posthuma*) because of its anatomical and physiological semblance with the human intestinal roundworm parasite [27]. After collection from moist soil, all worms were washed with normal saline water to eradicate all faecal matter and accustomed with laboratory environment before experimentation. In this experiment, we have used freshly prepared standard and test solutions. Test samples of the experimented extracts were prepared at the concentrations of 20–75 mg/ml in Tween 20 (1%) solution diluted with normal saline. Nearly equal sized earthworms were divided into seven groups (consisting of six worms in each) and were released into 30 ml of experimental formulation. The first group received Tween 20 along with normal saline and was taken as control; group two was treated with reference drug albendazole at a concentration of 20 mg/ml and considered as standard. Groups 4–7 were treated with different solvent extracts of *H. fomes* in various concentration (20 mg/ml, 25 mg/ml, 50 mg/ml, and 75 mg/ml). Time of paralysis and time of death of the experimented worms were noted. Time for paralysis was considered when any kind of movement could not be perceived excluding when the worms were shaken robustly. Time of death was concluded and documented after discerning that the worms neither moved when shaken vigorously nor when dipped in warm water (50°C) followed with fading away of their body colors.

### 2.8. Screening of Insecticidal Activity

Insecticidal activity of the experimented samples was assayed against *Tribolium castaneum.* These insects were collected from the stock cultures of the Crop Protection and Toxicology Laboratory, Sher-e-Bangla Agricultural University, Bangladesh. To perform the experiment, the test samples were prepared into six different concentrations (2.5 mg/ml, 5 mg/ml, 10 mg/ml, 20 mg/ml, 40 mg/ml, and 50 mg/ml) by dissolving the extracts into respective solvents. Then, the solutions were poured separately on the lower part of individual 60 mm Petri dishes and stand them for a while in open air to evaporate solvents. Then, six insects were placed in each of the treated Petri dishes including the control and kept them all in a secured place at room temperature. Three replicates were set up for the treated and control solutions. The mortality of the insects was checked initially after 30 minutes from the beginning and then later 1, 2, 4, 8, 12, and 48 h of exposure and the data were taken. Here, we used a simple microscope to trace the natural movement of its organs. Often, we used a warm needle closer to the bodies (lack of movement) to confirm death. The mortality record of the adult *T. castaneum* was calculated by the following formula [28]:

(3) $$\begin{array}{c} \text{Pr}=\frac{\text{P}o-\text{Pc}}{100-\text{Pc}}\times 100. \end{array}$$

Pr is the percentage (%) of corrected mortality; Po is the observed mortality; Pc is the control mortality, sometimes called natural mortality.

### 2.9. Statistical Analysis

In this study, all the in vitro experiments were performed in three replicates. Statistical analysis was completed by using SPSS software version 19.0. The results were articulated as mean ± standard error of mean (SEM), and Student's *t*-test and one way ANOVA followed by Dunnett's post hoc multiple comparison test were used to determine the values of *P*. The outcomes beneath *P* < 0.05 were considered as significant.

## 3. Results

### 3.1. Phytochemical Screening

The phytochemical screening of the experimented extracts of *H. fomes* revealed the presence of alkaloid, carbohydrate, tannins, flavonoid, saponin, glycoside, steroid, phenol, and resin (Table 1).

### 3.2. Thrombolytic Activity

When we added 100 *μ*l streptokinase (30,000 IU) to the clots along with 90 minutes incubation at 37°C, it exhibited a significant (67.77 ± 9.78%; Table 2) clot lysis; whereas, clots treated with 100 *μ*l sterile distilled water (negative control) revealed only insignificant clot lysis (10.44 ± 1.72%; Table 2). Here, we found that all the experimented solvent extracts of *H. fomes* possess thrombolytic activity. Among them, ethanolic extract of leaf revealed the highest percentage of clot analysis (33.12 ± 7.52%; Table 2). In contrast, ethyl acetate extract of bark showed the lowest level of clot analysis (20.97 ± 6.97%; Table 2). The detailed statistical representation of the effective clot lysis percentage by negative control (sterile distilled water), positive control (streptokinase), and different extracts is given in supplementary Table S1.

### 3.3. In Vitro Antiarthritic Activity

The antiarthritic test of the extracts of *H. fomes* was conducted in 5 different concentrations (100–500 *μ*g/ml). The obtained results are given in Table 3. Besides, the detailed statistical data of the experiment for assessing the antiarthritic activity are given in supplementary Table S2. All plant samples exhibited noteworthy protection against protein denaturation at diverse concentrations (dose levels). The utmost percentage inhibition of protein denaturation was noticed for chloroform extract of root (63.28 ± 5.96% at 500 *μ*g/ml), whereas the lowest inhibition was observed for ethanolic extract of root (36.12 ± 5.77% at 500 *μ*g/ml).

### 3.4. Anthelmintic Activity

The results of the anthelmintic activity of various extracts of *H. fomes* as well as albendazole (reference drug) on earthworm *P. posthuma* are given in Table 4. Besides, the detailed statistical data of the experiment for assessing the anthelmintic activity are given in supplementary Table S3. The experimented crude extracts exhibited significant anthelmintic activity in different concentrations and revealed paralysis and death of earthworms in dose-dependent manner in comparison with the reference drug. The ethanolic extract of bark at a concentration of 75 mg/ml took less period to cause paralysis and petite extra time to cause the death of earthworms in comparison with the reference drug.

### 3.5. Screening of Insecticidal Activity

**Figure 1:**
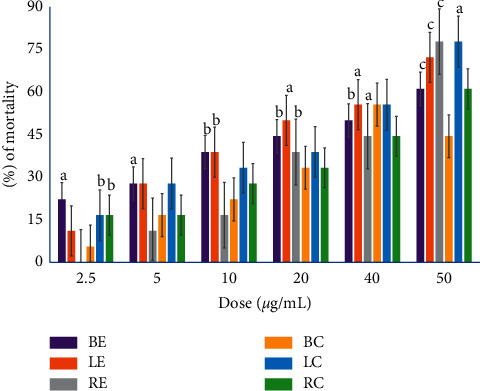
Insecticidal activity of chloroform (BC, LC, and RC) and ethanolic (BE, LE, and RE) extracts of bark, root, and leaf of *H. fomes*. The data were expressed as mean ± SEM. The *p* values were calculated from Student's *t*-test, where a, b, and c expressed *p* < 0.05, *p* < 0.01, and *p* < 0.001 vs. control, respectively.

**Figure 2:**
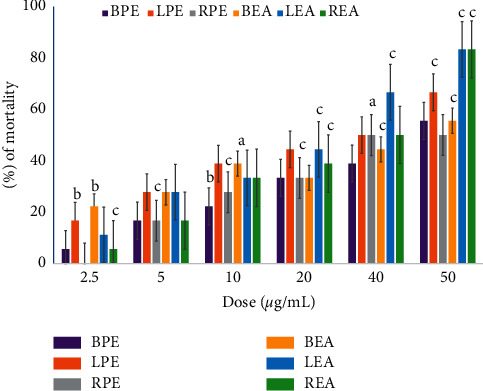
Insecticidal activity of ethyl acetate (BEA, REA, and LEA) and petroleum ether (BPE, RPE, and LPE) extracts of bark, root, and leaf of *H. fomes*. The data were expressed as mean ± SEM. The *p* values were calculated from Student's *t*-test, where a, b, and c expressed *p* < 0.05, *p* < 0.01, and *p* < 0.001 vs. control, respectively.

The insecticidal activity of *H. fomes* extracts is shown in Figures 1 and 2. Besides, the detailed statistical data of the experiment for assessing the insecticidal activity are found in supplementary Table S4. We noticed that their insecticidal potency increased in dose-dependent manner. The highest mortality rate was observed for ethanolic extract of leaf (73%).

## 4. Discussion

The interaction between humans and plants has long been described as one of the factors influencing human civilization, especially in the field of medicine [29, 30]. Still, 80% of people in developing countries prefer herbal formulations [31–33]. A phytopharmacological study has unwrapped a unique zone to discover plant-based medicines that are effective for the cure of different diseases and has been approved by the Food and Drug Administration [34]. *H. fomes* is a major mangrove species. Earlier studies reported the existence of alkaloids, tannins, polyphenols, steroids, saponins, glycosides, flavonoids, reducing sugars, gums, and carotenoids in different parts of *H. fomes* [17, 19], and these have been confirmed in parts by our own findings. Despite having huge potential, inadequate reports are available about its biological activities [17, 19–21]. These motivated us for further assay of different pharmacological activities of various parts of *H. fomes*. In this study, we have checked the in vitro thrombolytic, antiarthritic, anthelmintic, and insecticidal properties of ethanolic and its different solvent fractions of the experimented plant parts and found that they would be a great source to exploit new pharmaceuticals.

The rates of morbidity and mortality are noteworthy in developed countries due to different thromboembolic disorders such as pulmonary emboli, deep vein thrombosis, strokes, and heart attacks. Different atherothrombotic ailments such as myocardial or cerebral infarction may develop due to the growth of a thrombus that interrupts the blood flow through vessels. Even, sometimes in critical conditions, patients die due to embolism [35, 36]. Currently, numerous thrombolytic agents are clinically employed to dissolve the clots that have already formed in the blood vessels. Although some are natural medications and others are modified recombinant technology products to make them more effective and site-specific, they all have several life-threatening complications [37, 38] which inspired the scientists to search for a newer, safer, and effective thrombolytic remedy such as plant-derived drugs. Furthermore, recent epidemiologic studies with plant and natural products experimentally proved that natural thrombolytic/fibrinolytic agents have the competence to lessen the risk of thrombosis more than the synthetic ones [38, 39]. During a comparative study between positive and negative controls, we noticed that clot lysis did not occur when water was added to the clot; whereas, the addition of different fractions of the extract revealed significant clot lysis. Among the different fractions, LE exhibited the highest clot lysis (30.77 ± 4.09%). This activity may be owing to the presence of different bioactive components like alkaloids, flavonoids, and tannins which were previously claimed to possess clot lysis property [40].

Tissue protein denaturation is one of the most common causes of arthritic symptoms, which can result in the formation of autoantigens in some situations. Chemical exposure and heat can cause stress, which causes protein to denature and autoantigens to form, causing harm to the joint synovial membrane and cartilage. Denaturation of proteins can also be caused by changes in hydrogen, hydrophobic, disulphide, and electrostatic linkages [41, 42]. As a result, chemicals that limit protein denaturation would be advantageous in the creation of antiarthritic medications. In the current study, all extracts hindered BSA denaturation in dose-dependent manner. The utmost activity was noticed for chloroform extract of root at 500 *μ*g/ml (63.28 ± 5.96%). The inclusion of flavonoids and phenolic chemicals, which have previously been shown by several researchers, is thought to be responsible for the crude extracts' antiarthritic effect [43, 44].

Helminth infection is a severe problem in both humans and animals since it causes a chronic and debilitating illness that can lead to death and promote medication resistance in other diseases. Natural products such as medicinal plants must be studied to inhibit helminth infection because they produce novel bioactive compounds with no or few side effects, are easily accessible to people in developing countries, and, more importantly, have the best compatibility with human physiology than conventional drugs. Resistance to anthelmintics, toxicity, and increased concerns about medication residues in animal products have reignited interest in plant-based remedies [45, 46]. The outcomes of the anthelmintic test revealed that all the extracts possess moderate activity in dose-dependent manner. In this study, when we compared the time of paralysis and time of death of earthworms between plant extracts and standard, we observed that the result was nearly close to the reference drug. Potency of these extracts was inversely proportional to the time taken for paralysis/death of the worms. The ethanolic extract of bark was found to be most potent (paralysis at 39.67 ± 2.52 min and death at 49.67 ± 3.51 min) at a concentration of 75 mg/ml. Previous literature showed that tannins, alkaloids, and saponins possess anthelmintic properties [46, 47]. Tannin can attach to free proteins in the host animal's gastrointestinal tract or glycoprotein on the parasite's cuticle (earthworms) and cause death [48]. Saponins primarily work by irritating mucus membranes in a way that causes parasite death [47]. Additionally, tannins and alkaloids have a direct impact on the survivability of helminths' preparasitic stages and nervous system [46]. In phytochemical screening tests of extracts, we confirmed the abovementioned bioactive substances. As a result, our findings explain the plant's anthelmintic properties, although more research is needed to extract and describe the active ingredient.

Herbal preparations have recently received much attention as insecticidal substances due to their considerable potential. The mortality rate generated by each crude extract increased in a dose-dependent manner, according to our insecticidal investigation. We found that exposure time and dose were important factors in susceptibility. Plant extracts have previously been reported to have insect repellent, antifeedant, sterilizing, and toxic effects due to the presence of bioactive plant components, such as carbohydrates, saponins, phytosterol, phenol, flavonoids, and tannins, which have larvicidal action [49, 50]. As a result, polysaccharides, saponins, flavonoids, glycosides, and other secondary metabolites of the researched plant could help to explain the poisonous effect in the insects.

## 5. Conclusion

The current study assessed in vitro thrombolytic, antiarthritic, anthelmintic, and insecticidal activities of petroleum ether, chloroform, and ethyl acetate soluble fractions of ethanolic extract (leaf, bark, and root) of *H. fomes* Buch. Ham. In conclusion, it can be said that *H. fomes* has potent thrombolytic, antiarthritic, anthelmintic, and insecticidal activities based on the research. All the solvent fractions except the ethyl acetate fraction of the bark of *H. fomes* possess significant thrombolytic activity (*p* < 0.01). Chloroform extract of root showed the highest antiarthritic potentiality (63.28 ± 5.96% at 500 *μ*g/ml). The most insecticidal property was seen in the case of ethanolic extract of leaves (73%). However, further research is required for extensive chemical profiling to identify the bioactive components, which is mandatorily needed to delineate the exact mechanism of action of these bioactive crude fractions.

## Figures and Tables

**Table 1:** Screening of bioactive phytocompounds in different extracts of *H. fomes*.

Phytocompounds	Leaf				Root				Bark			
	LE	LEA	LC	LPE	RE	REA	RC	RPE	BE	BEA	BC	BPE
Carbohydrate	+	+	+	+	+	+	+	+	+	+	+	+
Glycoside	+	+	+	+	+	+	+	+	+	+	+	+
Tannin	+	+	+	+	+	+	+	+	+	+	+	+
Alkaloid	+	+	+	+	+	+	+	+	+	+	+	+
Saponin	+	+	+	+	+	+	+	+	+	+	+	+
Resin	+	+	+	+	+	+	+	+	+	+	+	+
Phenol	+	+	+	+	+	+	+	+	+	+	+	+
Flavonoid	+	+	+	+	+	+	+	+	+	+	+	+
Steroid	+	+	+	+	+	+	+	+	+	+	+	+
Fixed oil	−	−	−	−	−	−	−	−	−	−	−	−

+, present; −, negative.

**Table 2:** In vitro clot lysis activity of different extracts of *H. fomes.*

Sample	% of lysis
Water	10.44 ± 1.72
Streptokinase (standard)	67.77 ± 9.7^*∗∗*^
LE	33.12 ± 7.52^*∗*^
LPE	25.63 ± 4.63^*∗*^
LEA	26.39 ± 4.95^*∗*^
LC	27.76 ± 5.38^*∗*^
RE	24.28 ± 4.59^*∗*^
RPE	24.26 ± 4.35^*∗*^
REA	26.92 ± 6.11^*∗*^
RC	25.13 ± 4.81^*∗*^
BE	23.78 ± 4.77^*∗*^
BPE	27.03 ± 5.99^*∗*^
BEA	20.97 ± 6.97
BC	26.49 ± 5.62^*∗*^

Values are expressed as mean ± S.E.M. (*n* = 3). Data were analyzed by ANOVA followed by Student's *t*-test, ^*∗*^*p* < 0.01 and ^*∗∗*^*p* < 0.001.

**Table 3:** In vitro antiarthritic activity of *H. fomes* extracts in different concentrations.

Sample	% of inhibition				
	100 *μ*g/mL	200 *μ*g/mL	300 *μ*g/mL	400 *μ*g/mL	500 *μ*g/mL
Diclofenac sodium	61.63 ± 1.44	71.41 ± 1.49	78.13 ± 1.47	81.09 ± 3.08	87.45 ± 4.05
LPE	45.35 ± 2.9^*∗*^	46.82 ± 6.1^*∗*^	48.24 ± 5.82^*∗*^	49.69 ± 7.00^*∗*^	60.59 ± 6.66^*∗*^
LEA	42.82 ± 4.4^*∗*^	47.45 ± 4.5^*∗*^	49.99 ± 5.61^*∗*^	50.65 ± 7.76^*∗*^	59.53 ± 6.86^*∗*^
LC	36.73 ± 6.0^*∗*^	39.89 ± 6.4^*∗*^	45.98 ± 6.15^*∗*^	47.61 ± 5.06^*∗*^	48.93 ± 8.42^*∗*^
LE	41.19 ± 6.6^*∗*^	46.11 ± 5.0^*∗*^	48.15 ± 5.39^*∗*^	55.53 ± 6.02^*∗*^	62.08 ± 6.31^*∗*^
RPE	39.82 ± 5.5^*∗*^	43.97 ± 5.6^*∗*^	46.53 ± 5.53^*∗*^	54.04 ± 6.16^*∗*^	57.35 ± 8.73^*∗*^
REA	40.25 ± 5.1^*∗*^	43.97 ± 5.6^*∗*^	48.29 ± 7.61^*∗*^	52.34 ± 6.72^*∗*^	55.92 ± 8.31^*∗*^
RC	41.36 ± 4.6^*∗*^	50.53 ± 4.0^*∗*^	52.05 ± 6.58^*∗*^	53.75 ± 6.19^*∗*^	63.28 ± 5.96^*∗*^
RE	36.12 ± 5.7^*∗*^	38.06 ± 7.2^*∗*^	46.61 ± 6.51^*∗*^	49.02 ± 7.28^*∗*^	56.73 ± 7.75^*∗*^
BPE	38.85 ± 5.7^*∗*^	39.00 ± 7.3^*∗*^	41.84 ± 7.75^*∗*^	47.96 ± 6.94^*∗*^	55.33 ± 7.82^*∗*^
BEA	41.48 ± 5.3^*∗*^	45.69 ± 6.3^*∗*^	49.03 ± 6.31^*∗*^	56.21 ± 6.51^*∗*^	61.49 ± 6.73^*∗*^
BC	38.14 ± 6.0^*∗*^	42.87 ± 5.3^*∗*^	48.25 ± 6.73	50.84 ± 8.27^*∗*^	60.14 ± 7.54^*∗*^
BE	40.96 ± 5.18^*∗*^	44.11 ± 5.45	52.03 ± 6.5^*∗*^	53.78 ± 7.01^*∗*^	55.88 ± 7.72^*∗*^

Values are expressed as mean ± S.E.M (*n* = 3). Data were analyzed by ANOVA followed by Dunnett's *t*-test, ^*∗*^*p* < 0.01.

**Table 4:** Anthelmintic activity of *H. fomes* extracts in different concentrations.

Treatment	Conc. used (mg/ml)	Time taken for paralysis (min)	Time taken for death (min)
Control			
Standard	20	37.67 ± 1.53	45.33 ± 2.52

BE	25	50.67 ± 5.13^*∗*^	69.00 ± 7.55^*∗*^
	50	44.00 ± 2.00^*∗*^	54.33 ± 2.52^*∗*^
	75	39.67 ± 2.52	49.67 ± 3.51

LE	25	59.67 ± 8.08^*∗*^	67.00 ± 7.09^*∗*^
	50	48.33 ± 6.66^*∗*^	57.33 ± 3.05^*∗*^
	75	46.00 ± 5.29	52.67 ± 4.61

RE	25	52.33 ± 9.45	69.67 ± 8.02^*∗*^
	50	44.00 ± 7.81	54.00 ± 7.81
	75	42.00 ± 2.64	52.33 ± 4.16

BEA	25	50.33 ± 4.04^*∗*^	71.67 ± 8.50^*∗*^
	50	45.00 ± 2.00^*∗*^	55.33 ± 2.51^*∗*^
	75	45.33 ± 4.72	49.67 ± 2.52

LEA	25	60.67 ± 8.08^*∗*^	70.33 ± 8.38^*∗*^
	50	49.00 ± 6.93^*∗*^	54.00 ± 5.57
	75	48.33 ± 7.02	47.33 ± 3.78

REA	25	51.67 ± 9.61	70.00 ± 7.81^*∗*^
	50	45.00 ± 7.81	53.33 ± 5.13
	75	50.00 ± 8.01	46.67 ± 4.16

BC	25	51.00 ± 4.58^*∗*^	66.67 ± 7.50^*∗*^
	50	47.00 ± 6.56	58.00 ± 8.18
	75	45.67 ± 5.13	53.33 ± 4.93

LC	25	61.33 ± 7.64^*∗*^	68.67 ± 7.64^*∗*^
	50	51.67 ± 5.69^*∗*^	61.00 ± 4.58^*∗*^
	75	48.33 ± 8.03	52.33 ± 4.04

RC	25	51.67 ± 9.29	67.33 ± 6.81^*∗*^
	50	46.00 ± 7.81	56.00 ± 7.81
	75	41.33 ± 3.21	51.00 ± 7.55

BPE	25	53.00 ± 6.24^*∗*^	70.33 ± 8.50^*∗*^
	50	47.67 ± 3.51^*∗*^	64.00 ± 7.21^*∗*^
	75	46.33 ± 5.51	51.00 ± 3.60

LPE	25	61.00 ± 7.55^*∗*^	72.33 ± 6.51^*∗*^
	50	53.67 ± 5.69^*∗*^	69.00 ± 7.55^*∗*^
	75	49.33 ± 8.18	54.33 ± 2.08^*∗*^

RPE	25	56.67 ± 5.69^*∗*^	67.00 ± 6.56^*∗*^
	50	46.67 ± 7.23	62.00 ± 5.29^*∗*^
	75	49.33 ± 7.37^*∗*^	56.67 ± 7.23

Values are expressed as mean ± S.E.M (*n* = 3). Data were analyzed by ANOVA followed by Dunnett's *t*-test, ^*∗*^*p* < 0.01.

## Data Availability

The data used to support this study are included within the article and in the supplementary file.

## Supplementary Materials

Supplementary Materials The supplementary file containing four tables (Tables S1–S4) summarized the raw data involved in the study.
