# Supplementary material for: Phytochemical and Pharmacological Profiling of Heritiera fomes Buch. Ham. Deciphered Thrombolytic, Antiarthritic, Anthelmintic, and Insecticidal Potentialities via In Vitro Approach
Source: Evid Based Complement Alternat Med. 2022 Jul 18;2022:2594127. doi: 10.1155/2022/2594127 (PMC9345727; doi:10.1155/2022/2594127)
Supplement: Supplementary Materials — The supplementary file containing four tables (Tables S1–S4) summarized the raw data involved in the study. [file 2594127.f1.docx]

# **Phytochemical and pharmacological profiling of *Heritiera fomes* Buch. Ham deciphered thrombolytic, anti-arthritic, anthelmintic, and insecticidal potentialities via *in vitro* approach**

Farhana Alam Ripa ^1^, Md. Jamal Hossain ^2*^, Mst. Luthfun Nesa ^2^, Miss Sharmin Zahan ^2^, Saikat Mitra ^3^, Mohammad A. Rashid ^4^, Arpita Roy ^5*^, Saad Alghamdi ^6^, Mazen Almehmadi ^7^, Osama Abdulaziz ^7^

*^1^ Department of Pharmacy, Brac University, 41-Pacific Tower, Mohakhali, Dhaka-1212, Bangladesh*

*^2^ Department of Pharmacy, State University of Bangladesh, 77 Satmasjid Road, Dhanmondi, Dhaka-1205, Bangladesh*

*^3^ Department of Pharmacy, Faculty of Pharmacy, University of Dhaka, Dhaka-1000, Bangladesh*

*^4^ Department of Pharmaceutical Chemistry, Faculty of Pharmacy, University of Dhaka, Dhaka-1000, Bangladesh*

*^5^ Department of Biotechnology, School of Engineering & Technology, Sharda University, Greater Noida, India*

*^6^ Laboratory Medicine Department, Faculty of Applied Medical Sciences, Umm Al-Qura University, Makkah, Saudi Arabia*

*^7^ Clinical Laboratory Sciences Department, College of Applied Medical Sciences, Taif University, Taif Province, Kingdom of Saudi Arabia*

***Corresponding authors**

1. Md. Jamal Hossain; Email: [jamal.du.p48@gmail.com;](mailto:jamal.du.p48@gmail.com;) [jamalhossain@sub.edu.bd](mailto:jamalhossain@sub.edu.bd)
2. Arpita Roy; Email: [arbt2014@gmail.com](mailto:arbt2014@gmail.com)

**Table S1:** Thrombolytic activity of ethanol, petroleum ether, chloroform and ethyl acetate extracts of leaf, root and bark of *H. fomes* Buch. Ham.

|  | **Percentage (%) of clot lysis** | | | | | | | | | | | | | |
| --- | --- | --- | --- | --- | --- | --- | --- | --- | --- | --- | --- | --- | --- | --- |
|  | **Control** | **Standard** | **LE** | **LPE** | **LEA** | **LC** | **RE** | **RPE** | **REA** | **RC** | **BE** | **BPE** | **BEA** | **BC** |
| **1** | 8.9 | 58.33 | 24.53 | 26.32 | 21.83 | 25.77 | 24.09 | 20.23 | 21.76 | 29.99 | 21.43 | 27.89 | 15.65 | 30.7 |
| **2** | 10.11 | 67.11 | 36.32 | 20.68 | 25.68 | 23.66 | 19.78 | 23.68 | 25.33 | 20.38 | 20.67 | 20.66 | 18.4 | 20.11 |
| **3** | 12.3 | 77.86 | 38.52 | 29.88 | 31.66 | 33.86 | 28.96 | 28.87 | 33.66 | 25.01 | 29.23 | 32.54 | 28.86 | 28.67 |
| **Mean** | 10.4367 | 67.7667 | 33.12 | 25.63 | 26.39 | 27.76 | 24.28 | 24.26 | 26.92 | 25.13 | 23.78 | 27.03 | 20.97 | 26.49 |
| **SEM** | 1.723 | 9.781 | 7.522 | 4.639 | 4.953 | 5.384 | 4.592 | 4.349 | 6.106 | 4.806 | 4.737 | 5.986 | 6.969 | 5.620 |
| **P value** | | P<0.001 | P<0.01 | P<0.01 | P<0.01 | P<0.01 | P<0.01 | P<0.01 | P<0.01 | P<0.01 | P<0.01 | P<0.01 | **P>0.05** | P<0.01 |

**Note:** The ethanol, petroleum ether, chloroform and ethyl acetate extracts of leaf were tagged as LE, LPE, LC and LEA, respectively. Bark extracts were labeled as BE (Ethanolic), BPE (Petroleum ether), BC (Chloroform) and BEA (Ethyl acetate); whereas root extracts were marked as RE, RPE, RC and REA for ethanol, petroleum ether, chloroform and ethyl acetate solvents, correspondingly.

**Table S2:** Antiarthritic activity of ethanol, petroleum ether, chloroform and ethyl acetate extracts of leaf, root and bark of *H. fomes* Buch. Ham. (Dose = 100, 200, 300, 400 and 500 μg/mL)

|  | **Percentage (%) of inhibition** | | | | | | | | | | | | |
| --- | --- | --- | --- | --- | --- | --- | --- | --- | --- | --- | --- | --- | --- |
| **Dose (100 μg/mL)** | **Diclofenac**  **sodium** | LPE | LEA | LC | LE | RPE | REA | RC | RE | BPE | BEA | BC | BE |
| **1** | 60.11 | 41.38 | 37.7 | 30.11 | 35.73 | 34.11 | 38.97 | 38.27 | 30.92 | 32.23 | 41.99 | 38.11 | 37.23 |
| **2** | 63.56 | 48.56 | 45.87 | 38.09 | 41.33 | 40.46 | 35.89 | 39.14 | 35.11 | 41.65 | 35.85 | 32.08 | 38.78 |
| **3** | 61.23 | 46.11 | 44.9 | 41.99 | 46.51 | 44.88 | 45.88 | 46.68 | 42.33 | 42.67 | 46.59 | 44.23 | 46.87 |
| **Mean** | 61.633 | 45.35 | 42.82333 | 36.73 | 41.19 | 39.816 | 40.246 | 41.363 | 36.12 | 38.85 | 41.476 | 38.14 | 40.96 |
| **SEM** | 1.437042 | 2.9800 | 4.4633 | 6.0556 | 5.3913 | 5.4137 | 5.115 | 4.6248 | 5.7716 | 5.7557 | 5.388 | 6.0750 | 5.176553 |
| **P value** |  | P<0.01 | P<0.01 | P<0.01 | P<0.01 | P<0.01 | P<0.01 | P<0.00 | P<0.01 | P<0.01 | P<0.01 | P<0.01 | P<0.01 |
| **Dose**  **(200 μg/mL)** |  |  |  |  |  |  |  |  |  |  |  |  |  |
| **1** | 70.11 | 41.83 | 43.06 | 33.09 | 46.73 | 38.57 | 44.38 | 53.73 | 30.68 | 45.56 | 39.99 | 41.15 | 38.78 |
| **2** | 73.04 | 44.95 | 47.11 | 40.78 | 40.83 | 43.47 | 33.88 | 45.98 | 38.33 | 40.34 | 44.55 | 38.58 | 43.88 |
| **3** | 71.09 | 53.67 | 52.18 | 45.8 | 50.78 | 49.88 | 44.78 | 51.89 | 45.17 | 31.11 | 52.54 | 48.88 | 49.67 |
| **Mean** | 71.413 | 46.816 | 47.45 | 39.89 | 46.113 | 43.973 | 41.013 | 50.533 | 38.06 | 39.003 | 45.693 | 42.87 | 44.11 |
| **SEM** | 1.491 | 6.1367 | 4.5694 | 6.401 | 5.0035 | 5.6717 | 6.1808 | 4.0492 | 7.2487 | 7.3171 | 6.352 | 5.361 | 5.448 |
| **P value** | | P<0.01 | P<0.01 | P<0.01 | P<0.01 | P<0.01 | P<0.01 | P<0.01 | P<0.01 | P<0.01 | P<0.01 | P<0.01 | P<0.01 |
| **Dose (300 μg/mL)** |  |  |  |  |  |  |  |  |  |  |  |  |  |
| **1** | 76.56 | 42.13 | 43.98 | 40.19 | 40.83 | 40.18 | 40.93 | 45.09 | 40.32 | 33.17 | 42.48 | 41.66 | 52.95 |
| **2** | 78.34 | 48.88 | 50.9 | 45.31 | 49.86 | 49.18 | 47.79 | 52.9 | 46.17 | 44.23 | 49.56 | 47.99 | 45.05 |
| **3** | 79.49 | 53.72 | 55.09 | 52.44 | 53.77 | 50.24 | 56.14 | 58.16 | 53.33 | 48.11 | 55.06 | 55.11 | 58.09 |
| **Mean** | 78.13 | 48.243 | 49.99 | 45.98 | 48.153 | 46.533 | 48.286 | 52.05 | 46.606 | 41.836 | 49.033 | 48.253 | 52.03 |
| **SEM** | 1.476245 | 5.8211 | 5.6106 | 6.15242 | 6.6366 | 5.5276 | 7.6171 | 6.576329 | 6.5159 | 7.7522 | 6.3065 | 6.7288 | 6.568501 |
| **P value** | | P<0.01 | P<0.01 | P<0.01 | P<0.01 | P<0.01 | P<0.01 | P<0.01 | P<0.01 | P<0.01 | P<0.01 | P<0.01 | P<0.01 |
| **Dose (400 μg/mL)** |  |  |  |  |  |  |  |  |  |  |  |  |  |
| **1** | 79.91 | 48.35 | 41.99 | 52.11 | 48.95 | 52.98 | 49.99 | 49.81 | 55.11 | 49.25 | 54.67 | 48.33 | 45.98 |
| **2** | 78.77 | 43.45 | 52.97 | 42.13 | 56.87 | 48.48 | 47.11 | 50.55 | 50.99 | 40.46 | 50.61 | 44.11 | 55.79 |
| **3** | 84.58 | 57.26 | 56.99 | 48.58 | 60.77 | 60.66 | 59.93 | 60.88 | 40.95 | 54.17 | 63.35 | 60.07 | 59.57 |
| **Mean** | 81.08667 | 49.686 | 50.65 | 47.60667 | 55.53 | 54.04 | 52.3433 | 53.746 | 49.016 | 47.96 | 56.21 | 50.836 | 53.78 |
| **SEM** | 3.078544 | 7.0013 | 7.764457 | 5.060695 | 6.0228 | 6.1587 | 6.72619 | 6.1887 | 7.2833 | 6.9454 | 6.50811 | 8.2700 | 7.014421 |
| **P value** | | P<0.01 | P<0.01 | P<0.001 | P<0.01 | P<0.01 | P<0.01 | P<0.01 | P<0.01 | P<0.01 | P<0.01 | P<0.01 | P<0.01 |
| **Dose (500)** |  |  |  |  |  |  |  |  |  |  |  |  |  |
| **1** | 82.91 | 58.25 | 59.12 | 49.18 | 55.65 | 50.64 | 48.11 | 59.49 | 52.67 | 47.25 | 58.13 | 54.85 | 51.95 |
| **2** | 88.77 | 55.43 | 52.88 | 40.39 | 68.27 | 54.19 | 54.98 | 60.21 | 51.85 | 55.87 | 57.11 | 56.79 | 50.91 |
| **3** | 90.68 | 68.11 | 66.59 | 57.22 | 62.33 | 67.22 | 64.67 | 70.15 | 65.67 | 62.87 | 69.25 | 68.78 | 64.77 |
| **Mean** | 87.453 | 60.596 | 59.53 | 48.93 | 62.083 | 57.35 | 55.92 | 63.283 | 56.73 | 55.33 | 61.496 | 60.14 | 55.87667 |
| **SEM** | 4.0488 | 6.6577 | 6.86419 | 8.4177 | 6.3136 | 8.7300 | 8.3199 | 5.9575 | 7.7531 | 7.8239 | 6.7339 | 7.5450 | 7.719387 |
| **P value** | | P<0.01 | P<0.01 | P<0.01 | P<0.01 | P<0.01 | P<0.01 | P<0.01 | P<0.01 | P<0.01 | P<0.01 | P<0.01 | P<0.01 |

**Table S3:** Anthelmintic activity of ethanol, petroleum ether, chloroform and ethyl acetate extracts of leaf, root and bark of *H. fomes* Buch. Ham. (Dose = 25, 50 and 75 mg/mL)

|  |  | **Time (min) taken for paralysis** | | | | | | | | |
| --- | --- | --- | --- | --- | --- | --- | --- | --- | --- | --- |
|  |  | **25 mg/mL** | | | **50 mg/mL** | | | **75 mg/mL** | | |
|  | **Standard (albendazole-20mg/ml)** | **BPE** | **LPE** | **RPE** | **BPE** | **LPE** | **RPE** | **BPE** | **LPE** | **RPE** |
| **1** | 38 | 55 | 53 | 51 | 44 | 49 | 42 | 49 | 51 | 52 |
| **2** | 36 | 58 | 62 | 65 | 48 | 60 | 55 | 50 | 56 | 41 |
| **3** | 39 | 46 | 68 | 54 | 51 | 52 | 43 | 40 | 40 | 55 |
| **Mean** | 37.66666667 | 53 | 61 | 56.6666667 | 47.666667 | 53.666667 | 46.666667 | 46.33333 | 49 | 49.33333 |
| **SEM** | 1.527525232 | 6.244997998 | 7.5498344 | 7.3711148 | 3.5118846 | 5.6862407 | 7.2341781 | 5.507571 | 8.185353 | 7.371115 |
| **P value** |  | P<0.01 | P<0.01 | P<0.01 | P<0.01 | P<0.01 | P>0.05 | P>0.05 | P>0.05 | P<0.05 |
|  |  |  |  |  |  |  |  |  |  |  |
|  | **Standard (albendazole-20mg/ml)** | **BC** | **LC** | **RC** | **BC** | **LC** | **RC** | **BC** | **LC** | **RC** |
| **1** | 38 | 52 | 53 | 49 | 41 | 47 | 42 | 47 | 49 | 40 |
| **2** | 36 | 55 | 63 | 62 | 46 | 58 | 55 | 50 | 56 | 39 |
| **3** | 39 | 46 | 68 | 44 | 54 | 50 | 41 | 40 | 40 | 45 |
| **Mean** | 37.66666667 | 51 | 61.333333 | 51.6666667 | 47 | 51.666667 | 46 | 45.66667 | 48.33333 | 41.33333 |
| **SEM** | 1.527525232 | 4.582575695 | 7.6376262 | 9.29157324 | 6.5574385 | 5.6862407 | 7.8102497 | 5.131601 | 8.020806 | 3.21455 |
| **P value** |  | P<0.01 | P<0.01 | P>0.05 | P>0.05 | P<0.01 | P>0.05 | P>0.05 | P>0.05 | P>0.05 |
|  |  |  |  |  |  |  |  |  |  |  |
|  | **Standard (albendazole-20mg/ml)** | **BEA** | **LEA** | **REA** | **BEA** | **LEA** | **REA** | **BEA** | **LEA** | **REA** |
| **1** | 38 | 51 | 52 | 50 | 43 | 45 | 41 | 47 | 49 | 50 |
| **2** | 36 | 54 | 62 | 62 | 45 | 57 | 54 | 49 | 55 | 42 |
| **3** | 39 | 46 | 68 | 43 | 47 | 45 | 40 | 40 | 41 | 58 |
| **Mean** | 37.66666667 | 50.33333333 | 60.666667 | 51.6666667 | 45 | 49 | 45 | 45.33333 | 48.33333 | 50 |
| **SEM** | 1.527525232 | 4.041451884 | 8.0829038 | 9.60902354 | 2 | 6.9282032 | 7.8102497 | 4.725816 | 7.023769 | 8 |
| **P value** |  | P<0.01 | P<0.01 | P>0.05 | P<0.01 | P<0.05 | P>0.05 | P>0.05 | P>0.05 | P>0.05 |
|  |  |  |  |  |  |  |  |  |  |  |
|  | **Standard (albendazole-20mg/ml)** | **BE** | **LE** | **RE** | **BE** | **LE** | **RE** | **BE** | **LE** | **RE** |
| **1** | 38 | 52 | 51 | 49 | 42 | 44 | 40 | 42 | 48 | 41 |
| **2** | 36 | 55 | 61 | 63 | 44 | 56 | 53 | 40 | 50 | 40 |
| **3** | 39 | 45 | 67 | 45 | 46 | 45 | 39 | 37 | 40 | 45 |
| **Mean** | 37.66666667 | 50.66666667 | 59.666667 | 52.3333333 | 44 | 48.333333 | 44 | 39.66667 | 46 | 42 |
| **SEM** | 1.527525232 | 5.131601439 | 8.0829038 | 9.45163125 | 2 | 6.6583281 | 7.8102497 | 2.516611 | 5.291503 | 2.645751 |
| **P value** |  | P<0.01 | P<0.01 | P>0.05 | P<0.01 | P<0.05 | P>0.05 | P<0.01 | P>0.05 | P>0.05 |

**Table S4:** Insecticidal activity of ethanol, petroleum ether, chloroform and ethyl acetate extracts of leaf, root and bark of *H. fomes* Buch. Ham.

| **Treatment** | **Conc. Used (mg/ml)** | **% of mortality** |
| --- | --- | --- |
| Control | - | - |
| BE | 2.5 | Dead=1,2,1  16.67,33.33,16.67  MEAN=22.23 |
|  | 5 | Dead=2,2,1  33.33, 33.33,16.67  Mean=27.78 |
|  | 10 | Dead=2,3,2  33.33, 50,33.33  Mean=38.89 |
|  | 20 | Dead=2,3,3  33.33,50,50  Mean=44.44 |
|  | 40 | Dead=3,3,3  Mean=50 |
|  | 50 | Dead=4,3,3  66.67,50,66.67  Mean=61.11 |
| LE | 2.5 | Dead=1,1,0  16.67,16.67,0  Mean=11.13 |
|  | 5 | Dead=2,2,2  33.33,33.33,33.33  Mean=33.33 |
|  | 10 | Dead=2,2,3  33.33,33.33,50  Mean=38.89 |
|  | 20 | Dead=3,2,4  50,33.33,66.67  Mean=50 |
|  | 40 | Dead=4,3,4  66.67,33.33, 66.67  Mean=55.56 |
|  | 50 | Dead=5,4,4  83.33,66.67,66.67  Mean=72.22 |
| RE | 2.5 | Dead=0,0,0  Mean=0 |
|  | 5 | Dead=1,0,1  16.67,0,16.67  Mean=11.13 |
|  | 10 | Dead=1,1,1  16.67,16.67,16.67  Mean=16.67 |
|  | 20 | Dead=2,2,3  33.33,33.33,50  Mean=58.33 |
|  | 40 | Dead=2,2,4  33.33,33.33, 66.67  Mean=44.44 |
|  | 50 | Dead=4,5,5  Mean=44.44, 83.33,83.33  Mean=70.37 |
| REA | 2.5 | Dead=1,0,0  Mean=5.56 |
|  | 5 | Dead=1,1,1  Mean=16.67 |
|  | 10 | Dead=2,2,2 |
|  | 20 | Dead=2,2,3 |
|  | 40 | Dead=3,3,3 |
|  | 50 | Dead=4,4,4 |
| BEA | 2.5 | Dead=1,2,1 |
|  | 5 | Dead=2,2,1 |
|  | 10 | Dead=2,3,3 |
|  | 20 | Dead=3,2,3 |
|  | 40 | Dead=3,4,3 |
|  | 50 | Dead=5,4,4 |
| LEA | 2.5 | Dead=1,0,1 |
|  | 5 | Dead=1,2,1 |
|  | 10 | Dead=2,2,2 |
|  | 20 | Dead=3,2,3 |
|  | 40 | Dead=3,3,3 |
|  | 50 | Dead=4,4,4 |
| RC | 2.5 | Dead=0,1,2  0, 16.67, 33.33  Mean=16.67 |
|  | 5 | Dead=1,1,1  Mean=16.67 |
|  | 10 | Dead=2,1,2  33.33, 16.67, 33.33  Mean=27.77 |
|  | 20 | Dead=2,2,2  Mean=33.33 |
|  | 40 | Dead=3,3,2  Mean=44.44 |
|  | 50 | Dead=4,3,3  Mean=55.56 |
| BC | 2.5 | Dead=0,0,1  Mean=5.56 |
|  | 5 | Dead=1,1,1  Mean=16.67 |
|  | 10 | Dead=2,0,2  33.33, 0,33.33  Mean=22.22 |
|  | 20 | Dead=2,2,2  Mean=33.33 |
|  | 40 | Dead=4,2,4  44.44,33.33,44.44  Mean=39.39 |
|  | 50 | Dead=2,3,3  33.33, 50,50  Mean=44.44 |
| LC | 2.5 | Dead=1,1,1  Mean=16.67 |
|  | 5 | Dead=1,2,2  16.67, 33.33, 33.33  Mean=27.78 |
|  | 10 | Dead=3,0,3  50, 0, 50  Mean=33.33 |
|  | 20 | Dead=2,2,3  33.33, 33.33, 50  Mean=38.89 |
|  | 40 | Dead=3,3,4  50, 50, 44.44  Mean=48.15 |
|  | 50 | Dead=4,5,5  44.44, 83.33, 83.33  Mean=70.37 |
| LPE | 2.5 | Dead=1,1,1  Mean=16.67 |
|  | 5 | Dead=2,2,0  Mean=22.22 |
|  | 10 | Dead=2,2,3  Mean=38.87 |
|  | 20 | Dead=2,3,3  Mean=38.89 |
|  | 40 | Dead=3,3,3  Mean=50 |
|  | 50 | Dead=4,4,4  Mean=66.67 |
| BPE | 2.5 | Dead=1,0,0  Mean=5.56 |
|  | 5 | Dead=1,1,1  Mean= 16.67 |
|  | 10 | Dead=2,0,2  Mean=22.22 |
|  | 20 | Dead=2,2,2  Mean= 33.33 |
|  | 40 | Dead=3,2,3  Mean= 38.89 |
|  | 50 | Dead=3,3,4  Mean=48.15 |
| RPE | 2.5 | Dead=0,0,0  Mean=0 |
|  | 5 | Dead=1,1,1  Mean=16.67 |
|  | 10 | Dead=2,1,2  Mean=27.78 |
|  | 20 | Dead=2,2,2  Mean=33.33 |
|  | 40 | Dead=2,3,4  Mean=50 |
|  | 50 | Dead=1,4,4  Mean=50 |
